# Polarization of THP-1-Derived Macrophage by Magnesium and MAGT1 Inhibition in Wound Healing

**DOI:** 10.1055/s-0043-1770114

**Published:** 2023-08-02

**Authors:** Mun Ho Oh, JaeHyuk Jang, Jong Hun Lee

**Affiliations:** 1 Eulji Medi-Bio Research Institute, Eulji University, Seoul, Republic of Korea; 2 Department of Plastic and Reconstructive Surgery, Nowon Eulji Medical Center, School of Medicine, Eulji University, Seoul, Republic of Korea

**Keywords:** wound healing, magnesium, macrophage, differentiation, polarization

## Abstract

**Background** Macrophages play a major role in wound healing and prevent infection from the outside. Polarization conversion of macrophages regulates aspects of inflammation, and two macrophages, M1 (classically activated) and M2 (alternatively activated), exist at both ends of broad-spectrum macrophage polarization. Thus, we aimed to investigate whether macrophage polarization can be artificially regulated. To this end, MgSO4 and small-interfering RNA (siRNA) targeting magnesium transport 1 (MAGT1) were used to investigate the effects of intracellular magnesium (Mg2+) concentrations on the differentiation of macrophages in vitro.

**Methods** THP-1 derived macrophages maintained in a culture medium containing 5 mM MgSO4 and siRNA to inhibit the expression of MAGT1. As comparative groups, THP-1 derived macrophages polarized into M1 and M2 macrophages by treatment with M1, M2 inducer cytokine. The polarization status of each group of cells was confirmed by cell surface antigen expression and cytokine secretion.

**Results** We found that MgSO4 treatment increased CD163 and CD206, similar to the effect noted in the M2 group. The expression of CD80 and HLA-DR was increased in the group treated with MAGT1 siRNA, similar to the effect noted in the M1 group. Functional assays demonstrated that the group treated with MgSO4 secreted higher levels of IL-10, whereas the MAGT1 siRNA-treated group secreted higher levels of IL-6 cytokines. Additionally, the conditional medium of the Mg2+ treated group showed enhanced migration of keratinocytes and fibroblasts.

**Conclusion** Mg2+ can help to end the delay in wound healing caused by persistent inflammation in the early stages.

## Introduction

Wound healing is a complex series of processes that starts with a wound, but can continue into a diseased state for months or years. The whole process is dynamic and can be divided into inflammatory, fibroblastic, and maturation stages. 1 A prolonged inflammatory phase delays the next wound healing stage. Destroyed tissue debris, foreign substances, and necrotic tissue in this stage must be cleaned up by acute and chronic inflammatory cells before the next stage of healing begins. 2 Macrophages are one such cell derived from mononuclear cells in blood circulation. When mononuclear cells are attracted to a damaged area by chemotactic agents such as lymphocyte-derived chemotactic factor, N-formylemthiony-leucylphenylalanine, and collagen fragments, the mononuclear cells consume tissue debris, which triggers their transformation into macrophages. Simultaneously, local macrophages are also recruited. 3 Diversity and flexibility are two typical characteristics of macrophages. 4 5 Two macrophages, M1 (classically activated macrophages) and M2 (alternatively activated macrophages), exist at both ends of broad-spectrum macrophage polarization. 6 7 8 M1 macrophages have proinflammatory characteristics, defend against invading bacteria, and play a major role in the maintenance of homeostasis. 9 In contrast, M2 macrophages are involved in anti-inflammatory responses and tissue remodeling. It is well known that lipopolysaccharides (LPS) can induce macrophage polarization to the M1 phenotype, whereas interleukin 4 (IL-4) can induce macrophage polarization to M2. Recently, several studies have reported that macrophages are affected by various factors that alter their phenotype and affect their function. 10 The polarization shift of macrophages is of growing interest in regulating the initiation, generation, and resting phases of inflammatory diseases.

Magnesium (Mg ^2+^ ) is the most abundant divalent cation in living cells. 11 The critical interaction between phosphate and Mg ^2+^ makes Mg ^2+^ ions essential for the basic nucleic acid chemistry of all cells of living organisms. 12 13 More than 300 enzymes, including all enzymes that use or synthesize adenosine triphosphate (ATP), and those that use other nucleotides to synthesize DNA and RNA, require Mg ^2+^ ions for catalysis. ATP molecules are commonly found in chelates containing Mg ^2+^ ions. 14 In addition, Mg ^2+^ alters calcium (Ca ^2+^ ) and potassium (K ^+^ ) channels, and significantly affects the modification of intracellular ion dynamics and signaling. 15 The Mg ^2+^ transporter 1 (MAGT1) family is a group of magnesium transporters that are part of the transporter–opsin–G protein–coupled receptor (TOG) superfamily. MAGT1 has high selectivity for Mg(2 + ) and its possible involvement in cellular functions reaching far beyond magnesium homeostasis and MAGT1 functions as a second messenger that binds magnesium to intracellular effector receptor activation in certain cells. 16 In this study, MgSO _4_ and small-interfering RNA (siRNA) targeting MAGT1 were used to investigate the effect of intracellular magnesium concentration on the differentiation of macrophages. SiRNAs are part of the class of noncoding RNAs that repress their target mRNAs through their incorporation into the RNA-induced silencing complex.

In this study, we aimed to observe whether Mg ^2+^ can be used as a material for dressing preparations and whether it can affect wound healing by regulating macrophage polarization. We observed how magnesium induces polarization of macrophages through the secretion of specific cytokines, which in turn affects migration of keratinocytes and fibroblasts.

## Methods

### Cell Culture

To study monocyte/macrophage function and mechanisms, the human leukemia monocyte cell line THP-1 has been widely used in many studies. 17 In this study, the THP-1 cell line was also used to induce macrophage differentiation. Human monocytic THP-1 cells were obtained from the Korean Cell Line Bank (Seoul) and were cultured in Roswell Park Memorial Institute medium (RPMI 1640, Thermo Fisher Scientific) supplemented with 10% fetal bovine serum (FBS; Thermo Fisher Scientific), 10-mM Hepes (Thermo Fisher Scientific), 1-mM pyruvate (Thermo Fisher Scientific), 2.5-g/L D-glucose (Merck), and 50-PM β-mercaptoethanol (Thermo Fisher Scientific) at 37°C in a humidified atmosphere of 5% CO _2_ .

THP-1 monocyte–derived macrophages were observed after 48-hour incubation in RPMI medium, which contained 150-nM phorbol 12-myristate 13-acetate (PMA, Sigma). PMA is a diester of phorbol. It is a potent tumor promoter often employed in biomedical research to activate the signal transduction enzyme protein kinase C. 18 PMA is stimuli commonly used to induce macrophage differentiation in monocytic cell lines. 19 M1 macrophages were polarized by incubation of THP-1-derived macrophages with 20 ng/mL of interferon gamma (IFN-γ) (R&D Systems) and 10 pg/mL LPS (Sigma). Macrophage M2 polarization was obtained by incubation of TH-1-derived macrophages with 20 ng/mL of IL-4 (R&D Systems) and 20 ng/mL of IL-13 (R&D Systems).

### Cell Viability (MTT Assay)

To determine an appropriate MgSO _4_ treatment concentration, a 3-(4,5-dimethylthiazol-2-yl)-2,5-diphenyl tetrazolium bromide (MTT) assay was performed to observe cytotoxicity. THP-1 cells were seeded in 24-well plates at 180,000 cells/well and cultured overnight. After 24 hours of treatment with different concentrations of MgSO _4_ , cells were incubated for 2 hours with 500 μL of MTT reagent (2.5 mg/mL of phosphate-buffered saline [PBS], Sigma) in a CO _2_ incubator. Then, the medium was removed and 1 mL of lysis buffer (SDS 30%/N, N-dimethylformamide 2:1, pH 4.7) was added to each well. Plates were incubated at 37°C and gently shaken at 70 rpm for 1 hour. The absorbance was then measured at 570 nm using a microplate reader.

### Apoptosis and Proliferation Assay

To compare the apoptosis patterns of M1, M2, MgSO _4_ , and macrophages induced by MAGT1-targeting siRNA, an apoptosis assay was performed. THP-1 cells were cultured to subconfluency and differentiated into macrophages by PMA treatment. PMA-induced macrophages were then treated with LPS and IFN-γ for the M1 assay; IL-4 and IL-13 for M2 assay; and MgSO _4_ and MAGT1–1 siRNA for 24 hours to polarize the macrophages. MAGT1 siRNA was synthesized and purchased from Thermo Fisher Scientific and transfected using Lipofectamine RNAiMAX reagent (Thermo Fisher). Transfection efficiency was determined by using Block-iT Fluorescent Oligo kit (Thermo Fisher). Annexin V–fluorescein isothiocyanate (FITC) and propidium iodide (PI) were used to detect apoptotic cells according to the manufacturer's protocol. Briefly, after washing twice in PBS, 10 ^5^ cells were resuspended in 500-μL annexin buffer. The cells were suspended in a labeling solution containing FITC-conjugated annexin V antibody and PI for 5 minutes and then analyzed by flow cytometry.

For the proliferation assay, carboxyfluorescein succinimidyl ester (CFSE, Thermo Fisher Scientific) labeling procedure was performed. CFSE stock solution was prepared immediately prior to use by adding the appropriate volume of Dimethyl sulfoxide (DMSO). When the cells are grown to the desired density on culture flasks, the CFSE stock solution was diluted in prewarmed (37°C) PBS to the working concentration (5 µM). The culture medium from the cells was removed and replaced with the working solution and was incubated for 20 minutes at 37°C. After incubation, the loading solution was removed and the cells were washed twice with culture medium containing 1% protein, and replace with fresh, prewarmed complete culture medium and replaced with fresh, prewarmed complete culture medium. Cell stimulation, incubation was performed 10 minutes later and analyzed by flow cytometry.” to “Cells were incubated for 10 minutes to allow the CFSE reagent to undergo acetate hydrolysis and the fluorescence intensity was analyzed by flow cytometry.

### Flow Cytometry Analysis

The expression of macrophage cell surface markers was analyzed by flow cytometry. Cells were suspended in PBS containing 2% FBS. To prevent nonspecific antibody binding, a fragment crystallizable block was used according to the manufacturer's protocol. After treatment with macrophage surface marker–specific fluorescently labeled CD11c CD86 antibody, cells were incubated for 30 minutes and then washed three times with ice-cold washing buffer. Unstained samples were prepared for cell size and granularity assessments, and an isotype antibody was used to classify the fluorescence value generated by nonspecific binding as negative. Data were collected and analyzed using Accuri C6 flow cytometer.

### Cytokine Analysis

THP-1 cells were seeded at a density of 1 × 10 ^5^ cells per well (six-well plate). After differentiation into macrophages by treatment with 150-nM PMA, cells were divided into four groups and then treated with LPS and IFN-γ for the M1 assay; IL-4 and IL-13 for M2 assay; and MgSO4 and MAGT1–1 siRNA for 24 hours to polarize the macrophages respectively. For cytokine secretion analysis, the supernatant was collected by centrifugation and cytokines (IL-6, tumor necrosis factor α [TNFα], IL-10) in the supernatant were measured using a specific enzyme-linked immunosorbent assay (ELISA) kit (Thermo Fisher Scientific), according to the manufacturer's protocol. Absorbance was measured at 450 and 570 nm with a microplate reader.

### Cell-Based Scratch Assay

To observe how the culture medium of polarized macrophages affects the migration of keratinocytes and fibroblasts, a cell-based scratch assay was performed. In brief, human immortalized keratinocytes cells or human dermal fibroblasts were seeded in a six-well plate and cultured up to a 90 to 100% confluence level. To block cell proliferation, mitomycin C (10 μg/mL; Sigma) was added to the culture medium. After making a wound line by scraping the upper surface of the cultured cells with a 200-μL micropipette tip, the culture medium was removed and washed with PBS. The cells were then cultured for 0, 12, and 24 hours in M1 medium, M2 medium, Mg ^2+^ -induced macrophage medium, and MAGT1 siRNA–treated macrophage medium, respectively. The wound line was evaluated at four different sites, and the cell spacing was measured for each wound line area using ImageJ software (National Institutes of Health, Bethesda, MD). Migrated cells were counted at four different sites in each wound line site to determine human dermal fibroblast spacing. 20

### Statistical Analysis

Data were assessed and analyzed using a two-tailed Student's *t* -test and one-way analysis of variance. Statistical significance was set at *p* < 0.01.

## Results

### Effects of MgSO _4_ Treatment on THP-1 Cell Viability

**Fig. 1:**
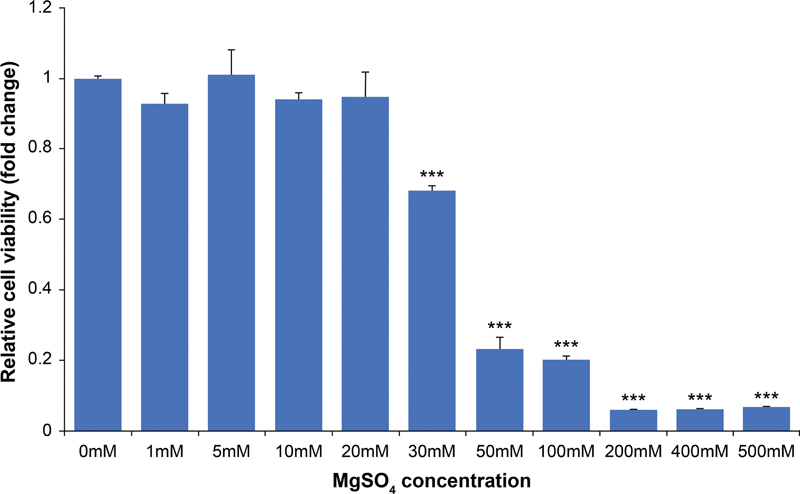
Dose-dependent cytotoxicity determined by MTT assay. Statistical significance of relative viability of THP-1 cells treated with various concentrations of MgSO _4_ (1–500 mM) compared with untreated THP-1 cells. Data are expressed as mean ± standard deviation of three independent experiments. Statistical data are significantly different at *** *p* < 0.001 as indicated by *t* -tests.

To evaluate the cytotoxicity of MgSO _4_ , THP-1 cells were treated with increasing concentrations of MgSO _4_ (0–500 mM). Cell proliferation was determined by MTT assay, and no cytotoxicity was observed up to 20-mM MgSO _4_ , whereas concentrations of more than 30 mM showed cytotoxic effects ( Fig. 1 ). Data are shown as mean ± SD (*** *p* < 0.001).

### MgSO _4_ Increases Intracellular Mg ^2+^ Levels in THP-1 Cells

**Fig. 2:**
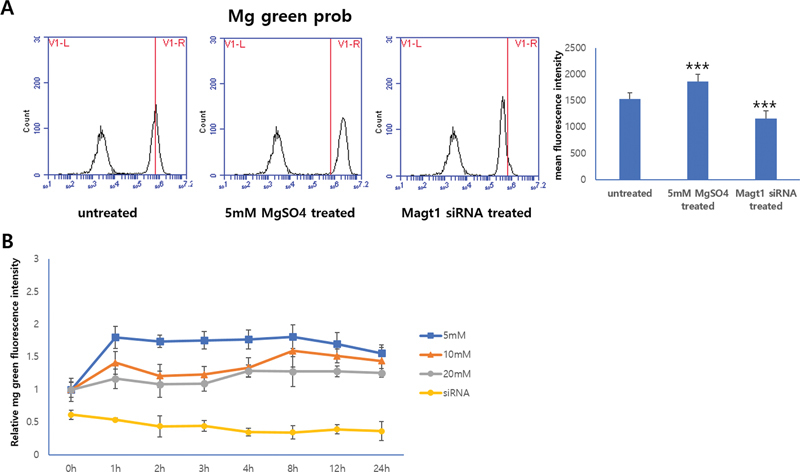
Differential expression of Magnesium Green probe in untreated, 5-mM MgSO _4_ -treated and MAGT1 siRNA treated THP-1 cells by flow cytometry. The histogram of the group treated with MgSO _4_ was shifted to the right, and the group treated with MAGT1 siRNA was shifted to the left ( **A** ) and difference in magnesium concentration with time-dependent manner ( **B** ). Data are expressed as mean of three independently repeated experiments ± SD (*** *p* < 0.001).

To evaluate the effect of MgSO _4_ on intracellular Mg ^2+^ levels, THP-1 cells were cultured in RPMI medium supplemented with 10% FBS or in the presence of 5-, 10-, and 20-mM MgSO _4_ . Using cell-permeant Magnesium Green probe staining ( Fig. 2A ), we found that intracellular Mg ^2+^ levels increased rapidly after 1 hour of supplementation with MgSO _4_ , and the intracellular Mg ^2+^ concentration remained high for 24 hours. On the other hand, the MAGT1 siRNA–treated group showed a lower intracellular Mg ^2+^ concentration than the MgSO _4_ treated group ( Fig. 2B ). These results support the concepts that extracellular Mg ^2+^ concentrations influence intracellular Mg ^2+^ levels and that cells can rapidly equilibrate Mg ^2+^ .

### Differentiation of PMA-Exposed THP-1 Cells into Macrophages

**Fig. 3:**
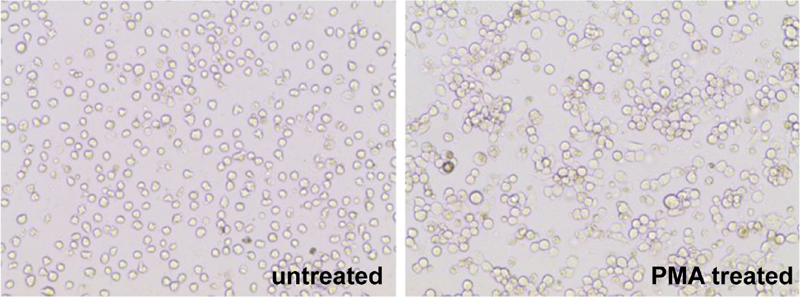
THP-1 cells exposed to phorbol 12-myristate 13-acetate are able to successfully differentiate into macrophages. The images of untreated and phorbol 12-myristate 13-acetate treated cells demonstrated measurable changes in morphology.

**Fig. 4:**
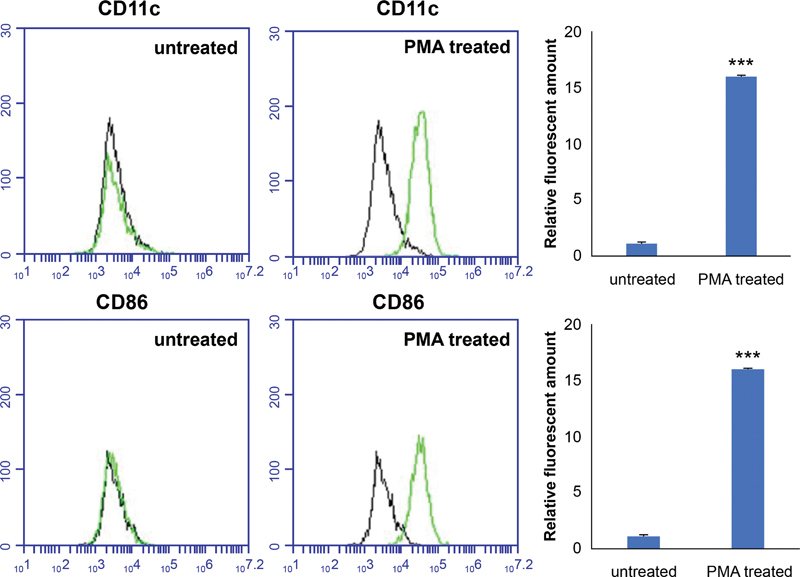
Characterization of the phenotype of THP-1 cells. The expression of macrophage markers (CD11c and CD86) on untreated and phorbol 12-myristate 13-acetate treated THP-1 cells was measured by flow cytometry analysis. The data are shown as mean ± SD (*** *p* < 0.001).

To stimulate the differentiation of THP-1-derived macrophages, 5-µM PMA was added to the culture medium for 48 hours ( Fig. 3 ). Cell morphology and expression patterns of CD11c and CD86-macrophage cell surface markers were used to determine the successful differentiation of THP-1-derived macrophages ( Fig. 4 ). The PMA-treated cells were larger, irregularly shaped, and adhered better to the culture flask surface.

### PMA-Induced Macrophages Were Polarized Similarly to Those of M2 and M1 Macrophages by MgSO4 and MAGT1 siRNA Treatment

**Fig. 5:**
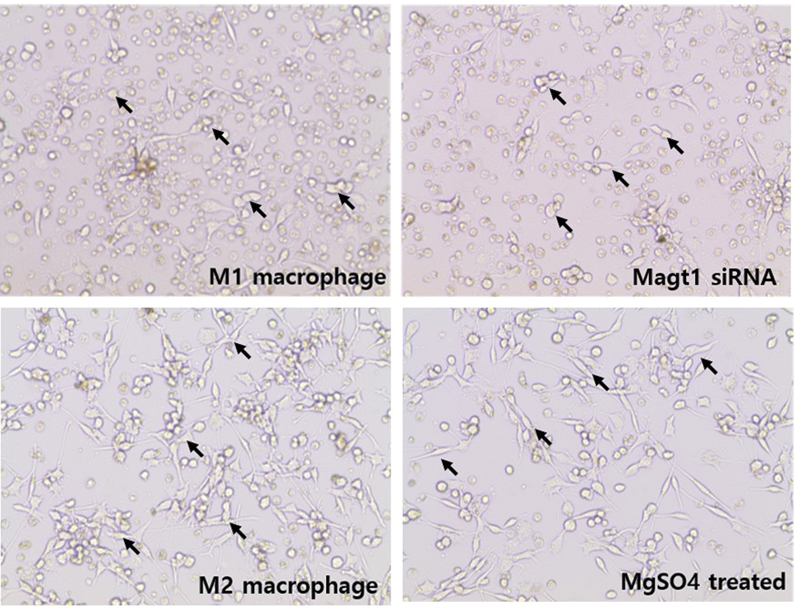
The effect of MgSO _4_ and MAGT1 siRNA on the morphologic change of phorbol 12-myristate 13-acetate–induced macrophages. Images of polarized macrophages derived from THP-1 cells showed significant morphological differences. *Arrows* indicate polarized macrophages in different conditions. M1 macrophages and MAGT1 siRNA–treated group showed enlarged cells and few dendrites. On the other hand, M2 macrophages and most cells of the magnesium-treated group showed dendritic appearance.

PMA-treated THP-1 cells were polarized to M1 and M2 and macrophages were exposed to MgSO _4_ and MAGT1 siRNA for 48 hours for comparison. In cytomorphology, M2 macrophages and most cells of the Mg ^2+^ -treated group showed a dendritic appearance. On the other hand, there were fewer dendritic cells in the M1 macrophages and cells in the MAGT1 siRNA–treated group ( Fig. 5 ).

**Fig. 6:**
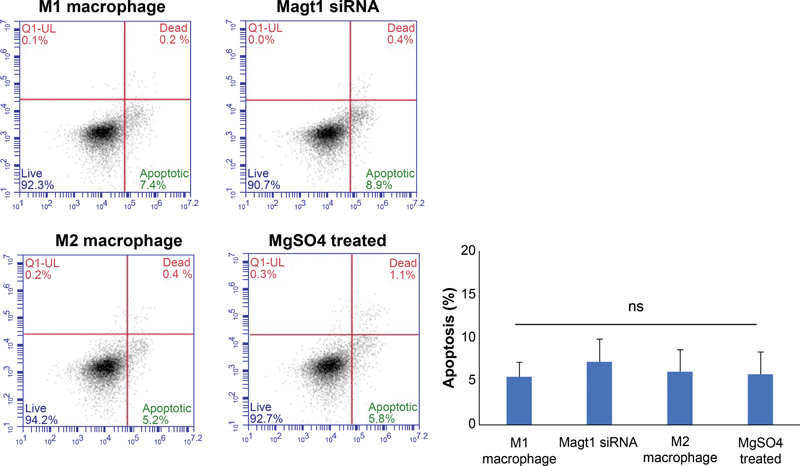
Apoptosis activity of MgSO _4_ and MAGT1 siRNA. Apoptotic cells of tested macrophages were assigned by annexin V–FITC/PI staining and investigated by flow cytometry. There were no measurable changes in each group. Data are expressed as mean of three independently repeated experiments ± SD.

Cells were stained with annexin V and PI to determine the level of cell apoptosis. No significant differences in apoptosis were observed in any of the four groups ( Fig. 6 ).

**Fig. 7:**
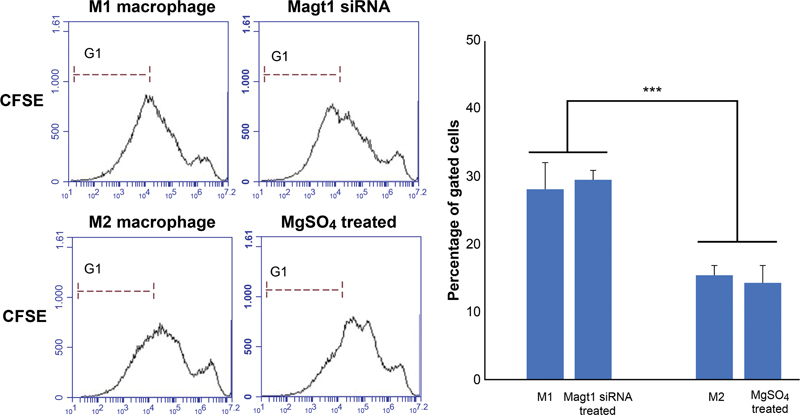
Phorbol 12-myristate 13-acetate–induced macrophages proliferation detected with carboxyfluorescein succinimidyl ester staining. Carboxyfluorescein succinimidyl ester low cells represent the cell population that has undergone proliferation. The histogram of M1 and the group treated with MAGT1 siRNA was shifted to the left, the histogram of M2 and the group treated with MgSO _4_ was shifted to the right. Data are shown as mean ± SD (*** *p* < 0.001).

The cells were fluorescently labeled on day 1 and stimulated with M1, M2 cytokine, MgSO _4_ , and MAGT1 siRNA. During proliferation, CFSE was equally distributed among “daughter cells,” whereas the overall fluorescence in each proliferating cell decreased ( Fig. 7 ). Cells treated with MgSO _4_ showed similar patterns to M2 macrophages, and cells treated with MAGT1 siRNA showed similar patterns to M1 macrophages.

### The Expression of Cell Surface Markers in the MgSO4-Treated Group Was Similar to That of M2 Macrophages; However, the MAGT1 siRNA-Treated Group Was Similar to That of the M1 Macrophages

**Fig. 8:**
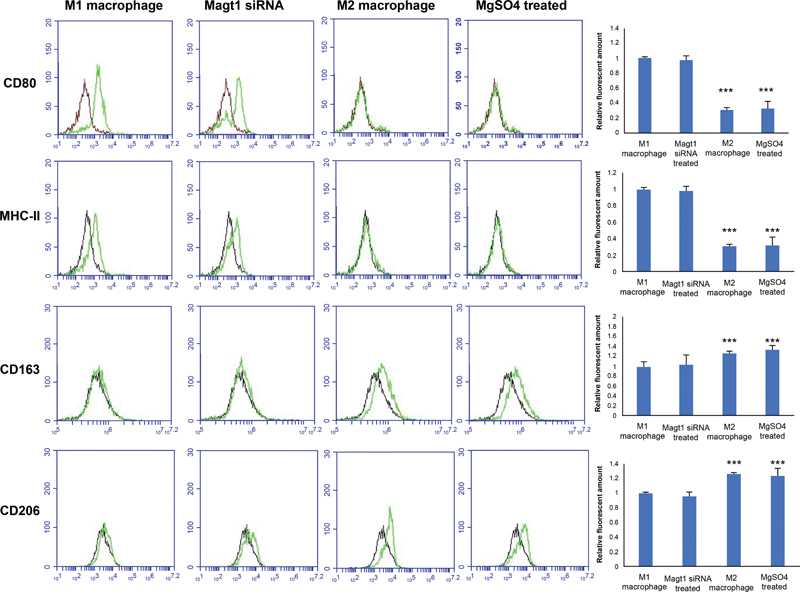
Characterization of the phenotype of polarized macrophages. The phenotype of polarized macrophages was defined by measuring the expression of cell surface markers with flow cytometry. M1 cell surface markers included MHC class II and CD80; M2 cell surface markers included CD163 and CD206. Data are expressed as mean of three independently repeated experiments ± SD (*** *p* < 0.001).

To clarify the polarization phenotype of MgSO _4_ and MAGT1 siRNA–induced macrophages, THP-1 cells were polarized to M1 and M2 as comparison targets. Cell surface markers were also analyzed ( Fig. 8 ). The expression levels of two M1 macrophage cell surface markers, major histocompatibility complex class II (MHC II) and CD80, and two M2 macrophage cell surface markers, CD163 and CD206, were analyzed using flow cytometry to observe the phenotype of MgSO _4_ and MAGT1 siRNA polarized macrophages. In the MgSO _4_ treatment group, the expression of CD163 and CD206 increased, but the expression of CD80 and MHC II did not. Based on the expression characteristics of these surface markers, these results indicated that the MgSO _4_ -treated group was similar to the phenotype of M2 macrophages, and the MAGT1 siRNA–treated group was similar to the phenotype of M1 macrophages.

### The Functions of MgSO _4_ -Treated Macrophages Are Similar to M2 Macrophages and MAGT1-Treated Macrophages Are Similar to M1 Macrophages

**Fig. 9:**
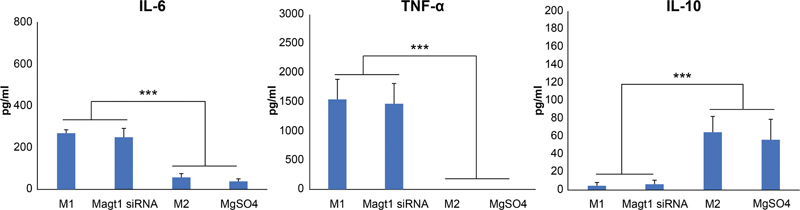
Functional analysis of polarized macrophages. The levels of TNFα, IL-6, and IL-10 in the cell culture supernatant were analyzed by ELISA. Data are expressed as mean of three independently repeated experiments ± SD (*** *p* < 0.001). IL, interleukin; TNF, tumor necrosis facror.

Normally, secretion of TNFα and IL-6 is characteristic of M1 macrophages, whereas the expression of anti-inflammatory cytokines such as IL-10 is characteristic of M2 cells. We evaluated the secretion pattern of these cytokines in the four groups of cells by ELISA ( Fig. 9 ). The results showed that M1 and MAGT1 siRNA–treated cells secreted higher levels of IL-6 and TNFα, whereas M2 macrophages and MgSO _4_ -treated cells secreted increased levels of IL-10.

**Fig. 10:**
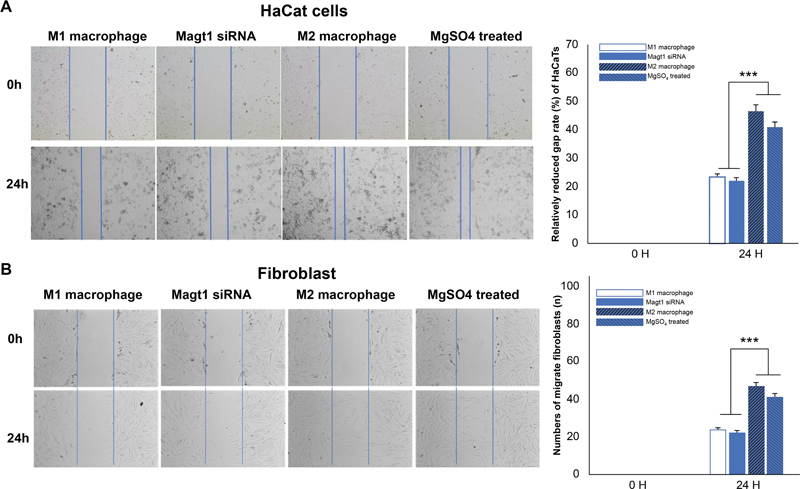
The effect of M1, M2 macrophage medium on migration of human keratinocyte (A) and human fibroblast (B) compare with MgSO4, MAGT1 siRNA–induced macrophage medium. Numbers indicate relatively reduced gap rate of keratinocyte and cell numbers of migrated fibroblasts. The data are shown as means ± SD (*** *p* < 0.001).

To further confirm the effect of macrophage polarization on the wound healing process, an in vitro wound healing assay was performed to mimic re-epithelialization through keratinocyte and fibroblast migration ( Fig. 10 ). Human immortalized keratinocyte cell migration was significantly inhibited in cells in M1 and MAGT1 siRNA–treated macrophage medium, which resulted in an extended wound closure period, while M2 and MgSO _4_ -treated cell medium significantly accelerated wound closure. These results demonstrate that elevated intracellular Mg ^2+^ levels appeared to function similarly to M2 macrophages.

## Discussion

The inflammatory response is a natural part of the wound healing process. Wounds heal as inflammatory cells catch harmful bacteria and regenerate the damaged area. 21 22 However, in patients with chronic inflammation, such as rheumatism, regeneration by macrophages (late inflammation) does not occur properly, 23 because existing inflammation in the patient's body disrupts the treatment pathway. In a healing wound process, macrophages represent the predominant cell type in the 3 to 5 days following injury. 24 Arriving at the site of injury several hours later than neutrophils, the main acute function of macrophages is to act as voracious phagocytes that cleanse the wound of all matrix and cellular debris, including fibrin and apoptotic neutrophils.

Macrophages also produce various cytokines, including growth and angiogenic factors that induce fibroblast proliferation and angiogenesis. 25 26 27 Macrophage polarization has been defined in at least two ways. M1 macrophages are considered to be effector cells in Th1 cellular immunity, while M2 macrophages support immunosuppression and wound repair. 28 Furthermore, M2 macrophages are divided into M2a, M2b, and M2c subtypes. 29 30 Due to the limitations of this project, we conducted a study with the main target of M2 macrophages differentiated by IL-4 and IL-13. To study macrophages, it is necessary to isolate human monocytes and differentiate them with cytokines such as IL-4, granulocyte macrophage colony-stimulating factor, and TNF-α. 4 31 32 However, a large amount of blood sample is required to isolate human monocytes, and even if the isolated monocytes are stored in a liquid nitrogen tank, the viability of the cells gradually decreases over time. One of the limitations is that human-derived monocytes cannot be significantly proliferated by general culture methods. 33 Considering these points, we paid attention to a cell line that can obtain a large amount of cells by general culture method and has properties similar to human-derived monocytes. As a human leukemia monocytic cell line, THP-1 is extensively used to study macrophage functions, mechanisms, signaling pathways, and nutrient and drug transport. 17 This cell line is considered a common model for estimating the regulation of monocyte and macrophage activities.

In this study, we investigated whether the differentiation of THP-1 cells could be artificially regulated through the difference in intracellular Mg ^2+^ concentrations. In this study, we tried to observe the effect of fine adjustment of magnesium concentration on cell differentiation under normal cell culture environment. Therefore, recognizing that the THP1 cell culture medium contains magnesium, an attempt was made to change the concentration of magnesium in such an environment. We first confirmed that MgSO _4_ or MAGT1 siRNA treatment affected the intracellular magnesium concentration of THP-1 cells. In THP-1 cells, apoptosis did not occur in a wide range of Mg ^2+^ concentrations; and when 5 mM Mg ^2+^ was used, the intracellular Mg ^2+^ concentration rose within 1 hour and decreased slightly over time, but maintained a higher concentration compared with the untreated group. In contrast, treatment with MAGT1 siRNA lowered intracellular Mg ^2+^ concentration. These results indicate that it may be possible to control the intracellular Mg ^2+^ concentration by treating the wound site with Mg ^2+^ and affecting the differentiation of macrophages. Cell morphology and expression patterns of CD11c and CD86-macrophage cell surface markers were used to determine the successful differentiation of THP-1-derived macrophages. 34 After the differentiated macrophages were treated with MgSO _4_ and MAGT1 siRNA, apoptosis and cell proliferation patterns were verified. There was no difference in apoptosis, though the cell proliferation patterns showed clear differences. These results suggest that differences in intracellular Mg ^2+^ concentrations can influence the polarization patterns of cells.

**Fig. 11:**
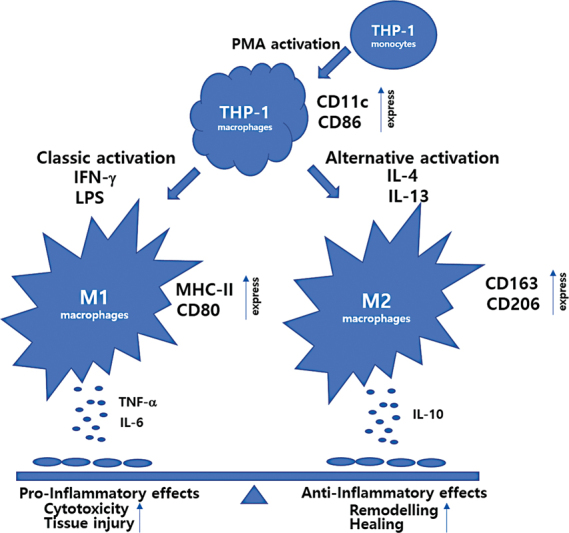
Schematic showing factors involved in THP-1 activation and polarization into M1 and M2 subtypes. THP-1 cells can be differentiated into macrophages (THP-1 macrophages) using 12-myristate 13-acetate (PMA). PMA-induced THP-1 macrophages can be further polarized into M1 (proinflammatory, classically activated) phenotype using LPS and IFN-γ or into M2 (anti-inflammatory, alternatively activated) using IL-4 and IL-13 treatment.

Macrophages derived from human monocytes undergo specific differentiation according to the tissue environment in which they are located. Phenotypically, MHC II, the CD68 marker, and costimulatory molecules CD80 and CD86 are known as M1 macrophage markers. In contrast, CD163 and CD206 are known markers characteristic of the M2 population. 4 35 36 37 In this study, it was also observed that MHC II and CD80 were increased in LPS- and IFN-γ-induced macrophages, and CD163 and CD206 were increased in IL-4- and IL-13-induced macrophages ( Fig. 11 ). Interestingly, the PMA-induced THP-1 cells treated with MgSO _4_ showed a phenotype similar to that of M2 macrophages, and the PMA-induced THP-1 cells treated with MAGT1 siRNA showed a phenotype similar to that of M1 macrophages. In general, M1 macrophages secrete TNFα and IL-6. In contrast, M2 cells are characterized by the secretion of anti-inflammatory cytokines, such as IL-10. Our observations of cellular secreted cytokine profiles indicate that MgSO _4_ -treated cells demonstrate cytokine expression patterns similar to that of M2 macrophages.

Macrophages are important immune effector cells of the innate immune system, provide an initial defense against microorganisms, and initiate and control the adaptive immune response. The ratio of M1 to M2 cells has been shown to have some predictive value in some diseases, such as rheumatoid arthritis, atopic dermatitis, and psoriasis. The data presented here indicate that this ratio also plays an important role in wound healing. The observation that different cell migration rates were observed when keratinocytes and fibroblasts were treated with the culture medium of MgSO _4_ -treated cells and the culture medium of MAGT1 siRNA–treated cells indirectly proves these hypotheses. Further research on the underlying mechanisms, using techniques such as 2D gel analysis or gene array, is necessary. The exact role of macrophage dendritic shape warrants further investigation, as well. In addition, because of the limitations of THP-1 cells as a cell line, verification using human mononuclear cells is required, and in vivo experiments are also required for further study.

Previous wound healing studies in our laboratory also focused on substances such as growth factors that act directly on keratinocytes. However, inspired by the gathering of large numbers of macrophages during the inflammatory phase of wounded tissue, this time we turned our attention to a different place to study whether macrophages could play a role in wound healing. Therefore, studies on whether macrophages have an effect or not have been performed ahead of studies on what mechanism affects them. For these reasons, this experiment is only a preliminary experiment to reveal the role of macrophages in wound healing. Certain substances such as fibroblast growth factor, transforming growth factor β, vascular endothelial growth factor, matrix metallopeptidase, and metalloproteinase inhibitor play an important role in wound healing. More experiments should be performed to reveal the interrelationship between macrophages and these substances, but limited research was conducted due to the limitations of support for this study. In addition, based on the results of this study, the next stage of research will be supplemented with a new thesis.

Together, the data presented here indicate that intracellular Mg ^2+^ concentrations had a significant effect on the differentiation of macrophages. When intracellular Mg ^2+^ was high, macrophage differentiation was polarized similar to that of the M2 type macrophages, and when the Mg ^2+^ concentration was low, differentiation was polarized similarly to that of the M1 type. Therefore, it is thought that using Mg ^2+^ , which can be easily added to wound dressing formulations, can help to end the delay in wound healing caused by persistent inflammation in the early stages. In addition, finding a key factor that regulates the differentiation of macrophages by Mg ^2+^ would be helpful in solving the problem of intractable wound healing.
